# Supplementary material for: Effect of Preoperative Inflammatory Diet on Clinical and Oncologic Outcomes Following Colorectal Cancer Surgery
Source: Nutrients. 2025 Apr 30;17(9):1522. doi: 10.3390/nu17091522 (PMC12074250; doi:10.3390/nu17091522)
Supplement: Supplementary file 1 [file nutrients-17-01522-s001.zip › nutrients-3558826-supplementary file S1.pdf]

Food Frequency Questionnaire	Name ______________	ID ______________

1. Respondent information: ◯ Self ◯ Proxy (Relationship to subject: __________)

2. In the past year, have there been any changes in the type or amount of food you consumed? (Changes in eating speed or time do not apply)
   ◯ No
   ◯ Yes (Changed from ________ months ago)
   ⇒ If your eating habits changed, please respond based on your dietary habits from the year before the change.

3. On average, how many meals did you have per day over the past year?
   ◯ 1 meal ◯ 2 meals ◯ 3 meals ◯ 4 meals ◯ Others (Irregular: __________)

※ The following questions pertain to your dietary habits over the past year. Please indicate how often and how much you ate each food item on average over the past year.

Rice and noodles (11)	How often did you eat this over the past year, on average?									Average Intake Amount
	Never	Month		Week			Day			
		1	2-3	1	2-4	5-6	1	2	3	

☞ 1 bowl: B1T (300 ml)
1 serving: standard restaurant serving size
Instant noodle 1 pack ≈ 120 g

1. Cooked rice	◯	◯	◯	◯	◯	◯	◯	◯	◯	◯ $^{1/2}$ bowl<br>◯ 1 bowl<br>◯ $1^{1/2}$ bowls<br>◯ 2 bowls
2. Cooked rice with other grains and legumes	◯	◯	◯	◯	◯	◯	◯	◯	◯	◯ $^{1/2}$ bowl<br>◯ 1 bowl<br>◯ $1^{1/2}$ bowls<br>◯ 2 bowls
3. Fried rice, cooked rice with assorted mixtures	◯	◯	◯	◯	◯	◯	◯	◯	◯	◯ $^{1/2}$ serving<br>◯ 1 serving<br>◯ $1^{1/2}$ servings
4. Rice rolled in laver	◯	◯	◯	◯	◯	◯	◯	◯	◯	◯ $^{1/2}$ roll<br>◯ 1 roll<br>◯ $1^{1/2}$ rolls<br>◯ 2 rolls
5. Curry and rice	◯	◯	◯	◯	◯	◯	◯	◯	◯	◯ $^{1/2}$ serving<br>◯ 1 serving

										○ $1^{1/2}$ servings
6. Instant noodles, instant cup noodles	○	○	○	○	○	○	○	○	○	○ $^{1/2}$ pack<br>○ 1 pack<br>○ $1^{1/2}$ packs
7. Noodles, Kalguksu, udong, Sujebi	○	○	○	○	○	○	○	○	○	○ $^{1/2}$ serving<br>○ 1 serving<br>○ $1^{1/2}$ servings
8. Cold noodles (naengmyeon, buckwheat noodles)	○	○	○	○	○	○	○	○	○	○ $^{1/2}$ serving<br>○ 1 serving<br>○ $1^{1/2}$ servings
9. Chinese black bean noodles, spicy seafood noodle soup	○	○	○	○	○	○	○	○	○	○ $^{1/2}$ serving<br>○ 1 serving<br>○ $1^{1/2}$ servings
10. Rice-cake soup, rice-cake and dumpling soup	○	○	○	○	○	○	○	○	○	○ $^{1/2}$ serving<br>○ 1 serving<br>○ $1^{1/2}$ servings
11. Dumpling (steamed or fried), Dumpling soup	○	○	○	○	○	○	○	○	○	○ $^{1/2}$ serving<br>○ 1 serving<br>○ $1^{1/2}$ servings

Soups and stews (11)	How often did you eat this over the past year, on average?									Average Intake Amount
	Never	Month		Week			Day			
		1	2-3	1	2-4	5-6	1	2	3	

☞ 1 bowl: D1B (250 ml)

1 cup: 200ml

12. Bean paste soup	○	○	○	○	○	○	○	○	○	○ $^{1/2}$ bowl<br>○ 1 bowl<br>○ $1^{1/2}$ bowls
13. Sea mustard soup	○	○	○	○	○	○	○	○	○	○ $^{1/2}$ bowl<br>○ 1 bowl<br>○ $1^{1/2}$ bowls
14. Beef soup, spicy beef soup	○	○	○	○	○	○	○	○	○	○ $^{1/2}$ bowl<br>○ 1 bowl<br>○ $1^{1/2}$ bowls
15. Dried alaska pollack soup	○	○	○	○	○	○	○	○	○	○ $^{1/2}$ bowl<br>○ 1 bowl<br>○ $1^{1/2}$ bowls
16. Kimchi stew	○	○	○	○	○	○	○	○	○	○ $^{1/2}$ cup<br>○ 1 cup<br>○ $1^{1/2}$ cups
17. Bean paste stew, fermented soybean	○	○	○	○	○	○	○	○	○	○ $^{1/2}$ cup<br>○ 1 cup

stew										○ $1^{1/2}$ cups
18. Bean curd stew, soft bean curd stew	○	○	○	○	○	○	○	○	○	○ $^{1/2}$ cup<br>○ 1 cup<br>○ $1^{1/2}$ cups
19. Frozen alaska pollack stew, spicy seafood stew	○	○	○	○	○	○	○	○	○	○ $^{1/2}$ bowl<br>○ 1 bowl<br>○ $1^{1/2}$ bowls
20. Beef born and meat potage	○	○	○	○	○	○	○	○	○	○ $^{1/2}$ bowl<br>○ 1 bowl<br>○ $1^{1/2}$ bowls
21. Potato and pork rib soup	○	○	○	○	○	○	○	○	○	○ $^{1/2}$ bowl<br>○ 1 bowl<br>○ $1^{1/2}$ bowls
22. Loach stew	○	○	○	○	○	○	○	○	○	○ $^{1/2}$ bowl<br>○ 1 bowl<br>○ $1^{1/2}$ bowls

Legumes, eggs, meats, and fish (26)	How often did you eat this over the past year, on average?									Average Intake Amount
	Never	Month		Week			Day			
		1	2-3	1	2-4	5-6	1	2	3	

☞ 1 cup: 200ml
1 tablespoon: 15ml
1 teaspoon: 5ml
1 egg = 6 quail egg
1 serving: standard restaurant serving size
½ raw squid ≈ 165g
Dried squid (whole) ≈ 80g

23. Bean curd, bean curd boiled in soy sauce	○	○	○	○	○	○	○	○	○	○ $^{1/4}$ cup<br>○ $^{1/2}$ cup<br>○ 1 cup
24. Soybean boiled in soy sauce	○	○	○	○	○	○	○	○	○	○ $^{1/2}$ tablespoon<br>○ 1 tablespoon<br>○ $1^{1/2}$ tablespoons
25. Fried egg, fried egg roll	○	○	○	○	○	○	○	○	○	○ $^{1/2}$ egg<br>○ 1 egg<br>○ 2 eggs
26. Boiled egg, steamed egg	○	○	○	○	○	○	○	○	○	○ $^{1/2}$ egg<br>○ 1 egg<br>○ 2 eggs
27. Grilled pork belly	○	○	○	○	○	○	○	○	○	○ $^{1/2}$ serving<br>○ 1 serving (150g)<br>○ 2 servings
28. Boiled pork	○	○	○	○	○	○	○	○	○	○ $^{1/2}$ cup

										○ 1 cup<br>○ $1^{1/2}$ cups
29. Stir-fried pork, grilled pork ribs, steamed pork ribs	○	○	○	○	○	○	○	○	○	○ $^{1/2}$ cup<br>○ 1 cup<br>○ $1^{1/2}$ cups
30. Sweet and sour pork, pork cutlet	○	○	○	○	○	○	○	○	○	○ $^{1/2}$ cup<br>○ 1 cup<br>○ $1^{1/2}$ cups
31. Grilled beef ribs	○	○	○	○	○	○	○	○	○	○ $^{1/2}$ serving<br>○ 1 serving (150g)<br>○ 2 servings
32. Stir-fried beef, steamed beef ribs	○	○	○	○	○	○	○	○	○	○ $^{1/2}$ cup<br>○ 1 cup<br>○ $1^{1/2}$ cups
33. Pork feet, grilled intestines, blood sausage	○	○	○	○	○	○	○	○	○	○ $^{1/4}$ cup<br>○ $^{1/2}$ cup<br>○ 1 cup
34. Ham, sausage	○	○	○	○	○	○	○	○	○	○ $^{1/8}$ cup<br>○ $^{1/4}$ cup<br>○ $^{1/2}$ cup
35. Dog meat stew	○	○	○	○	○	○	○	○	○	○ $^{1/2}$ serving<br>○ 1 serving (600ml)<br>○ $1^{1/2}$ servings
36. Korean traditional chicken soup	○	○	○	○	○	○	○	○	○	○ $^{1/2}$ serving<br>○ 1 serving (600ml)<br>○ $1^{1/2}$ servings
37. Fried chicken	○	○	○	○	○	○	○	○	○	○ 1 piece<br>○ 2 pieces<br>○ 3 pieces
38. Stir-fried chicken, chicken boiled with soy sauce	○	○	○	○	○	○	○	○	○	○ 1 cup<br>○ 2 cups<br>○ 3 cups
39. Grilled duck, stewed duck	○	○	○	○	○	○	○	○	○	○ $^{1/2}$ serving<br>○ 1 serving<br>○ $1^{1/2}$ servings
40. Mackerel, saury, Spanish mackerel	○	○	○	○	○	○	○	○	○	○ $^{1/8}$ cup<br>○ $^{1/4}$ cup<br>○ $^{1/2}$ cup
41. Hairtail, croaker	○	○	○	○	○	○	○	○	○	○ $^{1/8}$ cup<br>○ $^{1/4}$ cup<br>○ $^{1/2}$ cup
42. Anchovy, stir-fried anchovy	○	○	○	○	○	○	○	○	○	○ 1 teaspoon<br>○ 1 tablespoon<br>○ $^{1/4}$ cup

43. Fish ball (stir-fried, soup)	○	○	○	○	○	○	○	○	○	○ 1/4 cup<br>○ 1/2 cup<br>○ 1 cup
44. Squid (raw, dried)	○	○	○	○	○	○	○	○	○	※ raw or dried<br>○ 1/4 cup or 1/8 squid<br>○ 1/2 cup or 1/4 squid<br>○ 1 cup or 1/2 squid
45. Crab preserved in soy or spicy sauce	○	○	○	○	○	○	○	○	○	○ 1 teaspoon<br>○ 1 tablespoon<br>○ 1/4 cup
46. Shrimp (raw, grilled, fried)	○	○	○	○	○	○	○	○	○	○ 1 shrimp (40g)<br>○ 3 shrimps<br>○ 5 shrimps
47. Salted shrimp, squid and clam	○	○	○	○	○	○	○	○	○	○ 1/2 teaspoon<br>○ 1 teaspoon<br>○ 1 tablespoon
48. Sliced raw fish	○	○	○	○	○	○	○	○	○	○ 1/2 cup<br>○ 1 cup<br>○ 1 1/2 cups

Kimchi, vegetables, tubers, and seaweed (24)	How often did you eat this over the past year, on average?									Average Intake Amount
	Never	Month		Week			Day			
		1	2-3	1	2-4	5-6	1	2	3	

☞ 1 cup: 200ml

1 bowl: D1B (250 ml)

1 potato: C4 (113ml)

1 sweet potato: middle size (200ml=185g)

49. Korean cabbage kimchi	○	○	○	○	○	○	○	○	○	○ 1/8 cup<br>○ 1/4 cup<br>○ 1/2 cup
50. Other kimchi	○	○	○	○	○	○	○	○	○	○ 1/8 cup<br>○ 1/4 cup<br>○ 1/2 cup
51. Bean sprout (seasoned, soup), seasoned mung bean sprout	○	○	○	○	○	○	○	○	○	○ 1/8 cup<br>○ 1/4 cup<br>○ 1/2 cup
52. Seasoned spinach	○	○	○	○	○	○	○	○	○	○ 1/8 cup<br>○ 1/4 cup<br>○ 1/2 cup
53. Seasoned bellflower (boiled or not)	○	○	○	○	○	○	○	○	○	○ 1/8 cup<br>○ 1/4 cup<br>○ 1/2 cup

54. Pumpkin (seasoned, pan-fried)	○	○	○	○	○	○	○	○	○	○ 1/8 cup<br>○ 1/4 cup<br>○ 1/2 cup
55. Seasoned other vegetables (except for Q51-54)	○	○	○	○	○	○	○	○	○	○ 1/8 cup<br>○ 1/4 cup<br>○ 1/2 cup
56. Stir-fried mushroom	○	○	○	○	○	○	○	○	○	○ 1/8 cup<br>○ 1/4 cup<br>○ 1/2 cup
57. Seasoned green onion, seasoned Chinese chive	○	○	○	○	○	○	○	○	○	○ 1/8 cup<br>○ 1/4 cup<br>○ 1/2 cup
58. Cucumber (seasoned, raw)	○	○	○	○	○	○	○	○	○	○ 1/8 cup<br>○ 1/4 cup<br>○ 1/2 cup
59. Radish (seasoned, pickled, dried)	○	○	○	○	○	○	○	○	○	○ 1/8 cup<br>○ 1/4 cup<br>○ 1/2 cup
60. Vegetables salad	○	○	○	○	○	○	○	○	○	○ 1/4 cup<br>○ 1/2 cup<br>○ 1 cup
61. Pickled vegetable (pepper, garlic, sesame leaf)	○	○	○	○	○	○	○	○	○	○ 1/2 tablespoon<br>○ 1 tablespoon<br>○ 1 1/2 tablespoons
62. Raw vegetables (lettuce, sesame, Chinese cabbage, pumpkin leaf)	○	○	○	○	○	○	○	○	○	○ 5 leaves<br>○ 10 leaves<br>○ 15 leaves
63. Green pepper	○	○	○	○	○	○	○	○	○	○ 1 piece<br>○ 2 pieces<br>○ 3 pieces
64. Soybean paste sauce	○	○	○	○	○	○	○	○	○	○ 1 teaspoon<br>○ 2 teaspoons<br>○ 1 tablespoon
65. Grilled laver, raw laver, seasoned laver	○	○	○	○	○	○	○	○	○	○ 1/2 leaf<br>○ 1 leaf<br>○ 2 leaves
66. Seasoned green laver	○	○	○	○	○	○	○	○	○	○ 1 tablespoon<br>○ 1/4 cup<br>○ 1/2 cup
67. Stir-fried sea mustard stems	○	○	○	○	○	○	○	○	○	○ 1 tablespoon<br>○ 1/4 cup<br>○ 1/2 cup
68. Stir-fried	○	○	○	○	○	○	○	○	○	○ 1/4 cup

vegetable and noodles										○ 1/2 cup<br>○ 1 cup
69. Steamed corn, grilled corn	○	○	○	○	○	○	○	○	○	○ 1/2 corn<br>○ 1 corn<br>○ 1 1/2 corns
70. Steamed potatoes, grilled potatoes										○ 1/2 potato<br>○ 1 potato<br>○ 2 potatoes
71. Stir-fried potatoes, potatoes boiled with soy sauce	○	○	○	○	○	○	○	○	○	○ 1/8 cup<br>○ 1/4 cup<br>○ 1/2 cup
72. Sweet potatoes	○	○	○	○	○	○	○	○	○	○ 1/2 sweet potato<br>○ 1 sweet potato<br>○ 2 sweet potatoes

Milk (4)	How often did you eat this over the past year, on average?									Average Intake Amount
	Never	Month		Week			Day			
		1	2-3	1	2-4	5-6	1	2	3	
73. Milk (low fat, normal)	○	○	○	○	○	○	○	○	○	○ 1/2 cup<br>○ 1 cup<br>○ 1 1/2 cups
	73-1. Which type of milk did you usually consume?<br>○ Low fat ○ Normal ○ Both about equally									
74. Liquid type yogurt	○	○	○	○	○	○	○	○	○	○ small (65ml)<br>○ medium (130ml)<br>○ large (150ml)
75. Curd type yogurt	○	○	○	○	○	○	○	○	○	○ 1/2 serving<br>○ 1 serving (100g)<br>○ 2 servings
76. Soybean milk	○	○	○	○	○	○	○	○	○	○ 1/2 cup<br>○ 1 cup<br>○ 1 1/2 cups

77-0. During the past year, how frequently did you consume fruits on average?

Never	Month		Week			Day		
	1	2-3	1	2-4	5-6	1	2	3

※ The following questions ask about the average frequency and portion size of each fruit you consumed.

Please first indicate whether you usually consumed the fruit seasonally or year-round, and then answer the average frequency accordingly.

☞ 1 tomato: C5 size=30 cherry tomatoes
1 melon: O2 size
1 watermelon: F2*1cm size
1 peach: C5 size
grape, in cup
1 persimmon: C5 size
1 pear: C7 size
1 apple: C5 size
1 tangerine: C3 size

Fruits (12)	Seasonally?	Never	Month		Week			Day			Average Intake Amount
			1	2-3	1	2-4	5-6	1	2	3	
77. Banana	○ Yes<br>○ No	○	○	○	○	○	○	○	○	○	○ 1/2 piece<br>○ 1 piece<br>○ 2 pieces
78. Orange	○ Yes<br>○ No	○	○	○	○	○	○	○	○	○	○ 1/2 piece<br>○ 1 piece<br>○ 2 pieces
79. Strawberry	○ Yes<br>○ No	○	○	○	○	○	○	○	○	○	○ 5 pieces<br>○ 10 pieces<br>○ 15 pieces
80. Tomato, cherry tomato	○ Yes<br>○ No	○	○	○	○	○	○	○	○	○	○ 1/2 piece<br>○ 1 piece<br>○ 2 pieces
81. Melon	○ Yes<br>○ No	○	○	○	○	○	○	○	○	○	○ 1/3 piece<br>○ 1/2 piece<br>○ 1 piece
82. Watermelon	○ Yes<br>○ No	○	○	○	○	○	○	○	○	○	○ 1 piece<br>○ 2 pieces<br>○ 3 pieces
83. Peach	○ Yes<br>○ No	○	○	○	○	○	○	○	○	○	○ 1/2 piece<br>○ 1 piece<br>○ 2 pieces
84. Grape	○ Yes<br>○ No	○	○	○	○	○	○	○	○	○	○ 1/2 cup<br>○ 1 cup<br>○ 2 cups
85. Persimmon (raw, dried)	○ Yes<br>○ No	○	○	○	○	○	○	○	○	○	○ 1/2 piece<br>○ 1 piece<br>○ 2 pieces

86. Pear	○ Yes<br>○ No	○	○	○	○	○	○	○	○	○	○ 1/4 piece<br>○ 1/2 piece<br>○ 1 piece
87. Apple	○ Yes<br>○ No	○	○	○	○	○	○	○	○	○	○ 1/2 piece<br>○ 1 piece<br>○ 2 pieces
88. Tangerine	○ Yes<br>○ No	○	○	○	○	○	○	○	○	○	○ 1 piece<br>○ 2 pieces<br>○ 3 pieces

☞ 1 piece of pizza: 1/2 F3

1 serving of rice cake (in Q100): 10cm x 5cm x 4cm

Beverages, breads, and snacks (17)	How often did you eat this over the past year, on average?									Average Intake Amount
	Never	Month		Week			Day			
		1	2-3	1	2-4	5-6	1	2	3	
89. Coffee	○	○	○	○	○	○	○	○	○	○ 1 teaspoon<br>○ 2 teaspoons<br>○ 3 teaspoons
89-1. If you drank coffee more than 3 times a day, how many times on average? : ______ times per day										
89-2 Cream coffee	○	○	○	○	○	○	○	○	○	○ 1 teaspoon<br>○ 2 teaspoons<br>○ 3 teaspoons
89-3. Sugar coffee	○	○	○	○	○	○	○	○	○	○ 1 teaspoon<br>○ 2 teaspoons<br>○ 3 teaspoons
90. Green tea	○	○	○	○	○	○	○	○	○	○ 1/2 cup<br>○ 1 cup<br>○ 1 1/2 cups
91. Soft drink (cola, soda, fruit juice soda)	○	○	○	○	○	○	○	○	○	○ 1/2 cup<br>○ 1 cup<br>○ 1 1/2 cups
92. Fruit juice	○	○	○	○	○	○	○	○	○	○ 1/2 cup<br>○ 1 cup<br>○ 1 1/2 cups
93. Grain powder beverage, rice beverage	○	○	○	○	○	○	○	○	○	○ 1/2 cup<br>○ 1 cup<br>○ 1 1/2 cups
94. Sports drinks, amino acid beverages	○	○	○	○	○	○	○	○	○	○ 1/2 cup<br>○ 1 cup<br>○ 1 1/2 cups
95. Loaf bread	○	○	○	○	○	○	○	○	○	○ 1 slice<br>○ 2 slices

										○ 3 slices
95-1. Butter, margarine	○	○	○	○	○	○	○	○	○	○ 1 teaspoon<br>○ 2 teaspoons<br>○ 3 teaspoons
95-2. Jam	○	○	○	○	○	○	○	○	○	○ 1 teaspoon<br>○ 2 teaspoons<br>○ 3 teaspoons
96. Sweet red-beans buns, steamed sweet red-bean buns, Soboro buns	○	○	○	○	○	○	○	○	○	○ 1/2 serving<br>○ 1 serving<br>○ 2 servings
97. Castella, cake, muffin	○	○	○	○	○	○	○	○	○	○ 1/2 serving<br>○ 1 serving<br>○ 2 servings
98. Pizza	○	○	○	○	○	○	○	○	○	○ 1 piece<br>○ 2 pieces<br>○ 3 pieces
99. Hamburger, sandwich	○	○	○	○	○	○	○	○	○	○ 1/2 serving<br>○ 1 serving<br>○ 1 1/2 servings
100. Plain steamed rice-cake, steamed rice-cake with red bean, cubed rice-cake with soybean powder	○	○	○	○	○	○	○	○	○	○ 1/4 piece<br>○ 1/2 piece<br>○ 1 piece
101. Seasoned bar rice-cake	○	○	○	○	○	○	○	○	○	○ 1/2 cup<br>○ 1 cup<br>○ 1 1/2 cups
102. Snack	○	○	○	○	○	○	○	○	○	○ 1/2 cup<br>○ 1 cup<br>○ 1 1/2 cups
103. Cookie, cracker, biscuit	○	○	○	○	○	○	○	○	○	○ 3 pieces<br>○ 6 pieces<br>○ 9 pieces
104. Ice cream, ices	○	○	○	○	○	○	○	○	○	○ 1/2 scoop<br>○ 1 scoop<br>○ 2 scoops
105. Peanut	○	○	○	○	○	○	○	○	○	○ 1/4 cup<br>○ 1/2 cup<br>○ 1 cup

Alcohols (4)	How often did you eat this over the past year, on average?	Average Intake

☞ 1 cup of rice wine ≈ B4B

	Never	Month		Week			Day			Amount
		1	2-3	1	2-4	5-6	1	2	3	
106. Soju	○	○	○	○	○	○	○	○	○	○ 1/4 bottle<br>○ 1/2 bottle (180ml)<br>○ 1 bottle (360ml)<br>○ ___ bottles
107. Beer	○	○	○	○	○	○	○	○	○	○ 1/2 cup (250ml)<br>○ 1 cup (500ml)<br>○ 2 cups (1000ml)<br>○ ___ cups
108. Whiskey	○	○	○	○	○	○	○	○	○	○ 1 glass<br>○ 2 glasses<br>○ 3 glasses<br>○ ___ bottles
109. Rice wine	○	○	○	○	○	○	○	○	○	○ 1/2 cup<br>○ 1 cup<br>○ 2 cups<br>○ ___ cups

※ In addition to the food items listed above, were there any other foods (e.g., cereal, grilled eel, etc.) that you consumed more than once a week?

○ No

○ Yes (name of food item: ________ / frequency: ____ times per ____ weeks / amount: ________)
(name of food item: ________ / frequency: ____ times per ____ weeks / amount: ________)
(name of food item: ________ / frequency: ____ times per ____ weeks / amount: ________)

※ Thank you for your participation.
